# How much is social media worth? Estimating the value of Facebook by paying users to stop using it

**DOI:** 10.1371/journal.pone.0207101

**Published:** 2018-12-19

**Authors:** Jay R. Corrigan, Saleem Alhabash, Matthew Rousu, Sean B. Cash

**Affiliations:** 1 Department of Economics, Kenyon College, Gambier, OH, United States of America; 2 Department of Advertising + Public Relations, Michigan State University, East Lansing, MI, United States of America; 3 Sigmund Weis School of Business School, Susquehanna University, Selinsgrove, PA, United States of America; 4 Friedman School of Nutrition Science and Policy, Tufts University, Boston, MA, United States of America; Middlesex University, UNITED KINGDOM

## Abstract

Facebook, the online social network, has more than 2 billion global users. Because those users do not pay for the service, its benefits are hard to measure. We report the results of a series of three non-hypothetical auction experiments where winners are paid to deactivate their Facebook accounts for up to one year. Though the populations sampled and the auction design differ across the experiments, we consistently find the average Facebook user would require more than $1000 to deactivate their account for one year. While the measurable impact Facebook and other free online services have on the economy may be small, our results show that the benefits these services provide for their users are large.

## Introduction

In 2012, Facebook broadcasted its first-ever television advertisement, entitled “The Things That Connect Us” [1] The ad compared Facebook, in its utility and meaning, to a chair; an object that not only has functional value to individuals, but can also be at the heart of building and maintaining social relationships. The popularity of the network is in itself evidence that users value the service. If Facebook were a country, it would be the world’s largest in terms of population with over 2.20 billion monthly active users, 1.45 billion of whom are active on a daily basis [2], spending an average of 50 minutes each day on Facebook-owned platforms (e.g., Facebook, Messenger, Instagram) [3]. This means that, collectively, its users spend more than 100,000 years on Facebook each day. Despite concerns about loss of relevance due to declining personal posts by users [4], diminished interest in adoption and use by teens and young adults [5], claims about potential manipulation of its content for political purposes [6], and leaks that question the company’s handling of private user data [7], Facebook remains the top social networking site in the world and the third most visited site on the Internet after Google and YouTube [8].

Since its launch in 2004, Facebook has redefined how we communicate, maintain relationships, get information, manage public impressions of ourselves in a digital environment, and consume entertainment [9–13]. But compared to its cultural impact, Facebook’s macroeconomic impact is small. Facebook had 23,165 employees as of September 30, 2017 [2]. This is less than 1% the number employed by Walmart, the world’s largest private employer, and less than a third the number employed by Rite Aid, America’s 100th largest private employer [14]. Because Facebook’s users pay nothing for the service, Facebook does not contribute directly to gross domestic product (GDP), economists’ standard metric of a nation’s output [15]. In this context, it may seem surprising then that Facebook is the world’s fifth most valuable company [16] with a market capitalization of $541.56 billion in May 2018 [17], notwithstanding the dip in market capitalization following the most recent reputation crisis fueled by emerging news about Facebook’s role in the recent U.S. presidential elections [18]. In 2017, the company had $40.65 billion in revenues, primarily from advertising, and $20.20 billion in net income [19] while still offering the social networking service to its 2.20 billion users, at face value, for free. However, just because something is costless to a user does not imply that it has no value to that user. The question we pose here is just *how* valuable is Facebook to its users?

The answer to this question may shed light on the Solow Paradox, named for Robert Solow, the Nobel laureate economist who in 1987 observed, “What everyone feels to have been a technological revolution, a drastic change in our productive lives, has been accompanied everywhere … by a slowing-down of productivity growth, not a step up. You can see the computer age everywhere but in the productivity statistics” [20]. More concretely, the amount the average American worker produces grew by an average 2.8% per year in the post-war era through the 1960s, but grew by an average of 1.9% per year from 1970 to 2017 [21]. While a difference of less than a percentage point may seem inconsequential, through the power of compounding it means the average worker’s output doubles every 37 years rather than every 25 years. But what if these output measures severely underestimate the benefits information communication technologies like Facebook create for society more broadly? While it is generally the case that *price* is an underestimate of *value* to users, the distinction becomes even more marked when we try to consider the social value of services that are offered at no price to their users.

In this paper, we use experimental auctions to directly estimate the value U.S. users place on Facebook. Auction winners were paid to deactivate their account for as little as one hour or as long as one year. Because auction participants faced real financial consequences, they had an incentive to seriously consider what they would need to be compensated to go without the service for the time period specified. Though auction procedures and subject demographics varied across our four samples and different procedures, we consistently find that our 1,258 auction participants derive over $1000 of value annually on average from Facebook, reinforcing the idea that the computer age’s effect on society’s well-being is much larger than its effect on GDP.

## Theory

**Fig 1:**
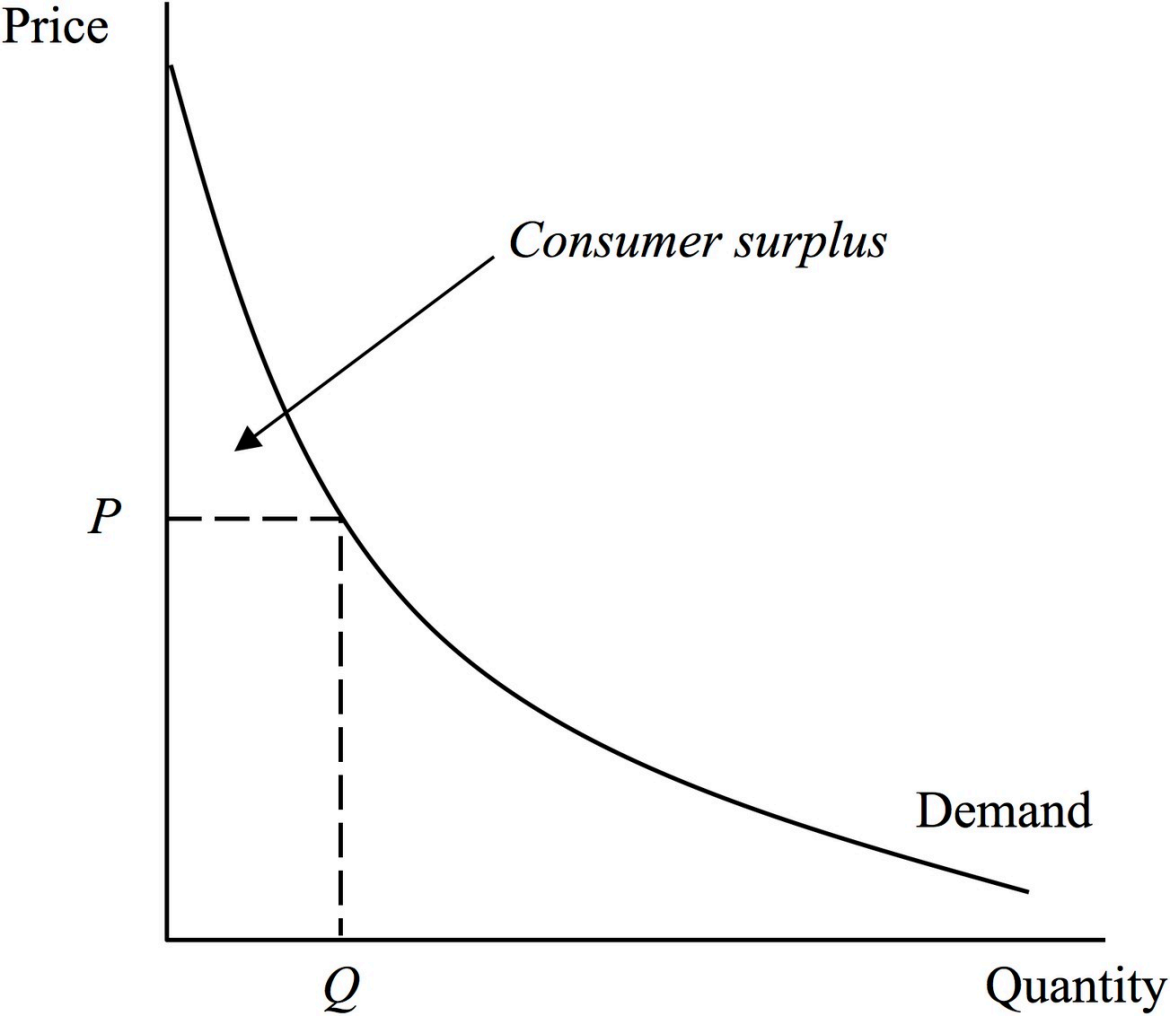
Consumer surplus is the sum of the amount users would be willing to pay for a service over what they actually pay for the service in the marketplace.

To measure the value of a product or service to consumers, economists would generally estimate *consumer surplus*. This is a measure of value equal to the difference between the most a consumer would be willing to pay for a service and the price she actually pays to use it. When considering all consumers, Fig 1 shows consumer surplus is the area under the demand curve, which shows consumers’ willingness to pay, and above the price; it is generally interpreted as consumers’ net benefit from being able to access a good or service in the marketplace [22]. GDP, by contrast, is the market value of all final goods and services produced domestically in a given year, and a market capitalization is often interpreted as the marketplace’s estimate of investors’ expectations of a company’s future flow of profits, discounted to the current time [23].

Free or low-cost online services may generate consumer surplus orders of magnitude greater than their measurable impact on GDP. Bapna, Jank, and Shmueli [24] found that eBay users received a median of $4 in consumer surplus per transaction in 2003, or $7 billion in total. Ghose, Smith, and Telang [25] found that Amazon’s used-book market generated $67 million in annual consumer surplus. Brynjolfsson, Hu, and Smith [26] found that the increased variety of books available on Amazon created $1 billion in consumer surplus in 2000.

Widening the lens to focus on the entire Internet, Greenstein and McDevitt [27] found that high-speed Internet access (as opposed to dial-up) generated $4.8 billion to $6.7 billion of consumer surplus in total between 1999 and 2006. Dutz, Orszag, and Willig [28] estimated that high-speed internet access generated $32 billion in consumer surplus in 2008 alone. Both studies used variations in the price households pay for high-speed internet to estimate demand and, by extension, consumer surplus. Goolsbee and Klenow [29] instead used the value of internet users’ time (i.e., their wages) to estimate the value derived from time spent online. The authors found that American households derived $2500 to $3800 in consumer surplus from the internet in 2004. According to the Pew Research Center [30], 54% of American adults had home internet access in 2004. Applying this percentage to America’s 112 million households in 2004 [31], Goolsbee and Klenow’s results suggest that Americans received $150 billion to $230 billion in consumer surplus.

## Methods and results

The authors of this study began as two separate teams that independently designed experimental auctions to estimate the economic value of Facebook. As a result, each auction has a slightly different design and advantages.

All of our experimental auctions use a Vickrey [32] second-price approach. In a typical experimental auction [33], participants bid to purchase a good or service. The highest bidder wins the auction and pays a price equal to the second-highest bid. This approach is designed such that participants’ best strategy is to bid their true willingness-to-pay, rather than underbid or overbid for strategic purposes [32, 34]. Each bid can therefore be interpreted as the maximum a participant would be willing to pay for a product, which in turn provides a means of estimating demand. Note that these are real auctions where winners actually purchase the product, in contrast to hypothetical experiments or surveys.

Because our study participants already had free access to Facebook, we could not ask people how much they would be willing to pay for access to the service. Instead, people bid for how much they would need in compensation to give up using Facebook. Economists have used these “willingness-to-accept” auctions to assess the value of mundane items such as pens and chocolate bars, but also more abstract or novel items such as food safety [35], goods free of genetically modified ingredients [36], the stigma associated with HIV [37], battery life in smartphones [38], and the payment people require to endure an unpleasant experience like tasting a bitter fluid [39] or listening to an annoying noise [40]. Unlike the vast majority of these applications, however, our approach is measuring the value of a product with which all participants are familiar and endowed prior to starting our study, minimizing concerns that the values we are measuring are ones that were first induced in the experimental setting rather than “homegrown” ones brought to the table by our subjects (e.g., [41]).

In this study, each bid can be interpreted as the minimum dollar amount a person would be willing to accept in exchange for not using Facebook for a given time period. The three auctions described below differ in the amount of time winners would have to go without using Facebook and, framing, and, importantly, sample selection. Across the three auctions, we use two samples of college students, a non-student community sample, and an online sample recruited using Amazon’s Mechanical Turk platform. While none of these samples is truly representative of Facebook’s users, paying participants to deactivate their accounts means we face a tradeoff between cost and representativeness that would not be present in a purely hypothetical survey.

### Auction 1 methods

One hundred twenty-two Facebook users on the campus of a Midwestern liberal arts college took part in Study 1, in groups of 10 to 12 participants at a time. Each received $20 for participating. Participants (Mean Age = 20.9, SD = 6.0; 64.2% Females) were given oral and written instructions on the auction mechanism, and then participated in a practice auction. For the practice auction, each participant received a pocket calculator and a pen, and submitted separate bids to sell each item in a second-price auction. After a monitor collected these bids, a volunteer rolled a die to determine which of the items would be sold. The lowest bidder for the selected item sold it to the monitor at a price equal to the second-lowest bid submitted. The purpose of this practice auction was to familiarize participants with the auction framework by showing them that they would place several bids but only one auction would be carried out, and that the low bidder in that binding auction would be paid the second-lowest bid for giving up something.

After the practice auction, participants bid in an auction to be paid to give up Facebook for one hour, one day, three days, and one week, with the understanding that one timeframe would be randomly chosen after all bids were collected, and that the selected timeframe would be binding on the low bidder.

In this auction, payment was tied to proof of deactivation. Participants understood that to receive the money for giving up Facebook, they would have to show the experimenter a page from their Facebook settings showing when they had deactivated and reactivated their account. Once this happened, the lowest bidder in each group was paid a price equal to the second-lowest bid offered in that group. This work was subject to human subjects review and approved by the Social Science/Behavioral/Education IRB at Michigan State University and the Institutional Review Board at Kenyon College, and written or online informed consent was obtained from all participants.

### Auction 1 results

Participants’ mean WTA for deactivating their Facebook account for one hour was $1.84 (*SD =* $3.41) with a median of $1.00. Mean WTA for deactivating for one day was $6.01 (*SD =* $12.21) with a median of $3.00. Mean WTA for deactivating for three days was $15.73 (*SD* = $34.51) with a median of $6.00. Mean WTA for deactivating for seven days was $38.83 (*SD* = $140.56) with a median of $15.00. Subtracting WTA for a one-hour deactivation from WTA for longer timeframes allows us to estimate net WTA, or willingness to accept after subtracting for the minor hassle of going through the process of deactivating and verifying deactivation. Table 1 presents summary statistics for WTA and net WTA, as well as estimated annual WTA based on linear extrapolations of net WTA. Ten of the eleven winners from the eleven auction sessions provided proof of deactivation and collected their payment.

**Table 1:** Summary statistics for Auction 1.

	Willingness to accept				Net willingness to accept (= X days– 1 hour)			Annualized net willingness to accept		
	1 hour	1 day	3 days	7 days	1 day	3 days	7 days	1 day	3 days	7 days
Mean	$1.84	$6.01	$15.73	$38.83	$4.17	$13.89	$37.00	$1,510.95	$1,676.15	$1,908.11
Std Dev	$3.41	$12.21	$34.51	$140.56	$9.20	$31.90	$138.68	$3,348.17	$3,868.35	$7,183.59
Min	$0.00	$0.00	$0.00	$0.00	$0.00	$0.00	$0.00	$0.00	$0.00	$0.00
Median	$1.00	$3.00	$6.00	$15.00	$2.00	$5.00	$13.25	$730.00	$608.33	$676.00
Max	$30.00	$100.00	$300.00	$1,500.00	$70.00	$285.00	$1,485.00	$25,550.00	$34,675.00	$77,220.00

Using random-effects regression analysis (not shown here), we find no evidence that gender, age, income, or the number of other social networking sites participants use affected WTA for Facebook. However, we find a statistically significant positive relationship between WTA and how frequently a participant posts status updates (p = 0.02) or uses Facebook to invite people to events (p = 0.02). Those who posted photos more often placed a lower value on Facebook (p = 0.02).

### Auction 1 discussion

Focusing on mean values, we find that net WTA for a three-day deactivation is more than three times net WTA for one day. Likewise, net WTA for a seven-day deactivation is more than seven times net WTA for one day. This suggests that, on average, the daily welfare lost from not having to access increases as the deactivation period increases. This is not true for median values for net WTA. In addition to being smaller in magnitude, median net WTA per day does not rise with the deactivation period.

Given the demographic homogeneity of our convenience sample of Facebook users, it is not surprising that our demographic variables are not statistically significant predictors of WTA. What is more surprising is that users who post photos more often place lower value on Facebook. There are, however, many other options for online photo sharing, most notably Instagram. It is possible that those who use Facebook primarily to share photos might be among the least invested in the specific form of online social networking offered by Facebook.

### Auction 2 methods

Participants in the second auction bid for how much they would need to close their Facebook account for an entire year. Participants were trained in the second-price auction through a tutorial and then a simulated auction in which participants were required to bid on how much they would need to be paid to sell the shoes they were wearing at the time of the study. Participants then took part in an actual second-price auction to close their Facebook accounts; they were warned that this was not hypothetical and that the experimenters would watch them deactivate their accounts before paying the second-lowest bid amount to the winner, and could then check their Facebook account throughout the year to ensure compliance.

After two pre-tests (*n* = 38) to ensure that the auction procedures were clear to participants, Auction 2 was replicated with two samples: a group consisting of 133 college students from a large Midwestern university (Mean Age = 20.44; SD Age = 1.20; 75.2% Female; 71.4% White/Caucasian) and another sample of 138 adults recruited via an online community subject pool at a large Midwestern college town (Mean Age = 23.67, SD Age = 8.28; 56.5% Female; 61.7% White/Caucasian). In addition to the amount received by auction winners, all student participants were compensated through credit in a student subject pool that could be used for participation points in cooperating classes, and all community pool participants received $10 for their participation in the study.

### Auction 2 results

Forty-one participants in the student sample declined to bid (bid “No”), and two participants bid more than $50,000, which for purposes of this analysis was considered to be a likely protest bid and outside of the population of interest. Removing these 43 responses leaves 90 responses for analysis. The average bid for deactivating Facebook for one year in the student sample was $2,076 (*SD =* $8,041), and the median bid was $200. Twenty-one participants in the community sample declined to bid, and two participants bid more than $50,000. Removing these 23 responses leaves 115 responses for analysis. In the community sample, the average bid was $1,139 (*SD* = $3,756), and the median bid was $100. A total of 21 winners received cash payment for their winning bids (4 additional participants won yet received $0), and prior to receiving payment were observed to deactivate their Facebook accounts.

### Auction 2 discussion

Though this auction differs from Auction 1 in terms of the timeframe of the deactivation, the verification requirement for payment, and the representativeness of the samples, all three mean annualized net WTA estimates from Auction 1 fall between the mean WTA estimates from Auction 2. This gives us greater confidence in our WTA estimates. There is a larger gap between median WTA estimates from Auctions 1 and 2. This is partly explained by the responses removed for analysis of our Auction 2 bids. Assuming participants who refused to bid and who submitted bids greater than $50,000 were from the top half of the value distribution, the median WTA bid from the student and community samples would be $600 and $200.

### Auction 3 methods

A third sample was recruited online through Amazon’s Mechanical Turk (MTurk), an “open online marketplace for getting work done by others,” where workers complete Human Intelligence Tasks (HITs) [40]. We posted a HIT, limited to residents of the United States, between February 2 and 12, 2015. Upon signing up for the study, participants were directed to the online questionnaire hosted on Qualtrics. Upon completion of the study, the 931 participants (Mean Age = 33.41; SD Age = 11.1; 53.1% Female) were credited 76 cents in exchange for their participation.

For the MTurk group, the in-person procedures described in Auction 2 were mimicked, but modified to fit online administration. There was still a tutorial on methods, but it was made to be a question-and-answer teaching session built to the online format. MTurk participants were grouped by the day, with the lowest bidder on a given day being paid the second-lowest bid in exchange for deactivating their account. There were 11 winners paid to deactivate their accounts (one for each day). Given that the study was conducted online, we did not collect the Facebook profile names for the winners and cannot confirm how many of these eleven deactivated their accounts.

### Auction 3 results

One hundred forty-five participants declined to bid, and 41 participants bid more than $50,000. Removing these 186 responses leaves 931 responses for analysis. The average bid in the MTurk sample was $1,921 (*SD* = $6,536), and the median bid was $100.

### Auction 3 discussion

Despite the potential for the online sample to have less engagement with the auction task than the in-person participants in the other samples, the quality of responses to the training questions and the similar distribution of results provide some reassurance that these participants understood the exercise and were taking it seriously [42]. Assuming participants who refused to bid and who submitted bids greater than $50,000 were from the top half of the value distribution, the median WTA bid would be $200.

## Conclusion

**Fig 2:**
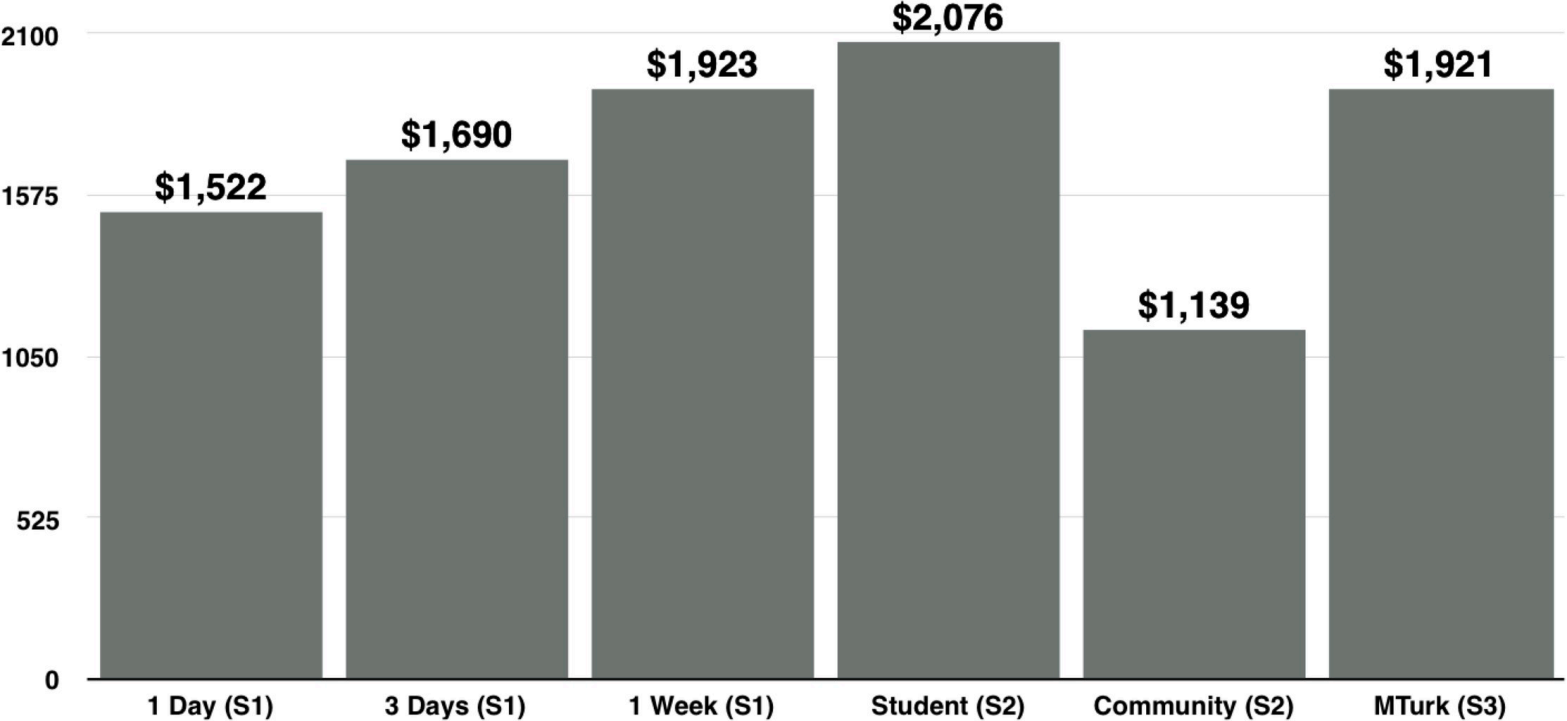
Mean bid amounts for a year off Facebook across samples and studies.

Fig 2 shows that the annual extrapolation for the one-week bid mirrors the findings from Auction 2 (Student sample; $2,076) and Auction 3 (MTurk sample; $1,921). The bid amount for the community sample in Auction 2 was relatively low. This could be a function of multiple factors. The community sample was considerably different in terms of socioeconomic make-up from the two homogenous student samples, yet not as representative as the MTurk sample. Nonetheless, across all three samples, the mean bid to deactivate Facebook for a year exceeded $1,000. Even the most conservative of these mean WTA estimates, if applied to Facebook’s 214 million U.S. users, suggests an annual value of over $240 billion to users.

As noted previously, Facebook reached a market capitalization of $542 billion in May 2018 [17]. At 2.20 billion active users in March 2018 [2], this suggests a value to investors of almost $250 per user, which is less than one fourth of the annual value of Facebook access from any of our samples. This reinforces the idea that the vast majority of benefits of new inventions go not to the inventors but to the users [43]. Further, our results provide evidence that online services can provide tremendous value to society even if their contribution to GDP is minimal. If the billions of people who use Facebook and other free online services derive anything close to $1000 per year in benefits, the productivity slowdown cited by Solow and others may not be reflected in a slowdown in the growth rate of welfare measures like consumer surplus. Many observers have commented on the difficulties of measuring productivity growth in great technological change [44]. While our current study does not offer a solution that can be broadly applied to address this challenge, it does present a methodology and results that provide important insight into the scale of the issue when considering the online revolution of our current era.

Concerns about data privacy, such as Cambridge Analytica’s alleged problematic handling of users’ private information, which are thought to have been used to influence the 2016 United States presidential election, only underscore the value Facebook’s users must derive from the service. Despite the parade of negative publicity surrounding the Cambridge Analytica revelations in mid-March 2018, Facebook added 70 million users between the end of 2017 and March 31, 2018 [45]. This implies the value users derive from the social network more than offsets the privacy concerns.

## References

[1] Stampler L. This Is Facebook's First Ever Major, Agency Created Ad—And It's Pretty Existential [Video]. Business Insider. 4 Oct 2012. Available from: http://www.businessinsider.com/this-is-facebooks-first-ever-major-ad-2012-10.

[2] Facebook. 2018. Available from: https://newsroom.fb.com/company-info/.

[3] Stewart J. Facebook Has 50 Minutes of Your Time Each Day. It Wants More. The New York Times. 5 May 2016. Available from: https://www.nytimes.com/2016/05/06/business/facebook-bends-the-rules-of-audience-engagement-to-its-advantage.html?_r=0.

[4] Armstrong P. Facebook Users Posted A Third Less Content In 2016 Than In 2015. Forbes. 14 Feb 2017. Available from: https://www.forbes.com/sites/paularmstrongtech/2017/02/14/facebook-users-posted-a-third-less-content-in-2016-than-in-2015/2/#1a6ff8c258ed.

[5] Ghosh S. Facebook really is losing teen users to Instagram and Snapchat. Business Insider. 22 Aug 2017. Available from: http://www.businessinsider.com/facebook-losing-teen-users-faster-to-instagram-and-snapchat-2017-8.

[6] Hern A. Facebook and Twitter are being used to manipulate public opinion–report. The Guardian. 19 Jun 2017. Available from: https://www.theguardian.com/technology/2017/jun/19/social-media-proganda-manipulating-public-opinion-bots-accounts-facebook-twitter.

[7] Seetharaman, Deepa. Facebook Suspends Data Firm That Helped Trump Campaign. The Wall Street Journal, Dow Jones & Company. 17 Mar 2018. Available from: www.wsj.com/articles/facebook-suspends-cambridge-analytica-for-failing-to-delete-user-data-1521260051?mod=article_inline&page=3&pos=14.

[8] The Top 500 Sites on the Web. Alexa Internet. 2018. Available from: https://www.alexa.com/topsites.

[9] Alhabash S, Chiang YH, Huang K. MAM & U&G in Taiwan: Differences in the uses and gratifications of Facebook as a function of motivational reactivity. Computers in Human Behavior. 6 2014; 35:423–30. 10.1016/j.chb.2014.03.033

[10] Alhabash S, Park H, Kononova A, Chiang YH, Wise K. Exploring the Motivations of Facebook Use in Taiwan. Cyberpsychology, Behavior, and Social Networking. 15 6 2012; 15(6):304–11. 10.1089/cyber.2011.0611 22703036

[11] Whiting A, Williams D. Why people use social media: a uses and gratifications approach. Qualitative Market Research: An International Journal. 2013; 16(4):362–9. 10.1108/QMR-06-2013-0041

[12] Mantymaki M, Najmul Islam AKM. The Janus face of Facebook: Positive and negative sides of social networking site use. Computers in Human Behavior. 8 2016; 61:14–26. 10.1016/j.chb.2016.02.078

[13] Seidman G. Self-presentation and belonging on Facebook: How personality influences social media use and motivations. Personality and Individual Differences. 2 2013; 54(3):402–7. 10.1016/j.paid.2012.10.009

[14] Fortune. 2017 [Available from: http://fortune.com/fortune500/list/filtered?sortBy=employees&first500.

[15] Brynjolfsson E, Saunders A. What the GDP Gets Wrong (Why Managers Should Care). Mit Sloan Manage Rev. 01 10 2009; 51(1):96-+.

[16] Forbes. 2018. The World’s Largest Public Companies. Available from: https://www.forbes.com/global2000/list/#header:marketValue_sortreverse:true. Accessed October 18, 2018.

[17] “Facebook Inc. Market Cap:” YCharts. 2018. Available from: https://ycharts.com/companies/FB/market_cap.

[18] Shen, Lucinda. Facebook Gains $21 Billion in Value as Mark Zuckerberg Testifies Before Congress. Fortune. 10 Apr 2018. Available from: http://fortune.com/2018/04/10/heres-why-facebook-just-gained-21-billion-in-value/

[19] Facebook. Facebook Reports Fourth Quarter and Full Year 2016 Results. 2017. Available from: https://investor.fb.com/investor-news/press-release-details/2018/Facebook-Reports-Fourth-Quarter-and-Full-Year-2017-Results/default.aspx.

[20] Solow RM. You’d Better Watch Out. New York Times. 12 July 1987.

[21] U.S. Bureau of Labor Statistics, Nonfarm Business Sector: Real Output Per Hour of All Persons [OPHNFB], retrieved from FRED, Federal Reserve Bank of St. Louis; https://fred.stlouisfed.org/series/OPHNFB, 22 October 2018.

[22] Just RE, Hueth DL, Schmitz A. The Welfare Economics of Public Policy: A Practical Approach to Project and Policy Evaluation: E. Elgar; 2004.

[23] Bodie Z, Kane A, Marcus AJ. Investments. Tenth edition. ed. New York: McGraw-Hill Education; 2014 1 volume (various pagings)

[24] Bapna R, Jank W, Shmueli G. Consumer Surplus in Online Auctions. Information Systems Research. 31 7 2008; 19(4):400–16. 10.1287/isre.1080.0173

[25] Ghose A, Smith MD, Telang R. Internet exchanges for used books: An empirical analysis of product cannibalization and welfare impact. Information Systems Research. 01 3 2006; 17(1):3–19. 10.1287/isre.1050.0072

[26] Brynjolfsson E, Hu Y, Smith MD. Consumer surplus in the digital economy: Estimating the value of increased product variety at Online booksellers. Management Science. 01 11 2003; 49(11):1580–96. 10.1287/mnsc.49.11.1580.20580

[27] Greenstein S, McDevitt RC. The broadband bonus: Estimating broadband Internet's economic value. Telecommunications Policy. 8 2011; 35(7):617–32. 10.1016/j.telpol.2011.05.001

[28] Dutz M A, Orszag J M, Willig R D. The Liftoff of Consumer Benefits from the Broadband Revolution. Review of Network Economics. 12 12 2012 11(4):1446–9022. 10.1515/1446-9022.1355

[29] Goolsbee A, Klenow PJ. Valuing consumer products by the time spent using them: An application to the Internet. American Economic Review. 5 2006; 96(2):108–13. 10.1257/000282806777212521

[30] Internet/Broadband Fact Sheet 2017. Pew Research Center, Internet & Technology. Available from: http://www.pewinternet.org/fact-sheet/internet-broadband/.

[31] Bureau USC. Current Population Survey, Annual Social and Economic Supplement. 2004. Available from: https://www2.census.gov/programs-surveys/demo/tables/age-and-sex/2004/age-sex-composition/2004gender_table5.xls.

[32] Vickrey W. Counterspeculation, Auctions, and Competitive Sealed Tenders. The Journal of Finance. 3 1961; 16(1):8–37. 10.1111/j.1540-6261.1961.tb02789.x

[33] Lusk JL, Shogren JF. Experimental Auctions: Methods and Applications in Economic and Marketing Research. Cambridge University Press; 2007 10.1017/CBO9780511611261

[34] Lucking-Reiley D. Vickrey auctions in practice: From nineteenth-century philately to twenty-first-century e-commerce. The Journal of Economic Perspectives. 2000; 14(3):183–92. 10.1257/jep.14.3.183

[35] Fox JA, Hayes DJ, Shogren JF. Consumer Preferences for Food Irradiation: How Favorable and Unfavorable Descriptions Affect Preferences for Irradiated Pork in Experimental Auctions. Journal of Risk and Uncertainty. 1 2002; 24(1):75–95. http://www.jstor.org/stable/41761057

[36] Rousu MC, Lusk JL. Valuing Information on GM Foods in a WTA Market: What Information is Most Valuable. Agbioforum. 2009; 2009 v.12 no.2 (no. 2):pp. 226–31.

[37] Hoffmann V, Fooks JR, Messer KD. Measuring and Mitigating HIV Stigma: A Framed Field Experiment. Economic Development and Cultural Change. 7 2014; 62(4):701–26. 10.1086/676145

[38] Hosio S, Ferreira D, Goncalves J, Berkel Nv, Luo C, Ahmed M, et al. Monetary Assessment of Battery Life on Smartphones. Proceedings of the 2016 CHI Conference on Human Factors in Computing Systems; San Jose, California, USA. 2858285: ACM; 2016. p. 1869–80.

[39] Coursey DL, Hovis JL, Schulze WD. The Disparity between Willingness to Accept and Willingness to Pay Measures of Value. The Quarterly Journal of Economics 01 8 1987; 102(3):679–90. 10.2307/1884223

[40] Ariely D, Loewenstein G, Prelec D. "Coherent arbitrariness": Stable demand curves without stable preferences. The Quarterly Journal of Economics. 01 2 2003; 118(1):73–105. 10.1162/00335530360535153

[41] Cherry TL, Frykblom P, Shogren JF, List JA, Sullivan MB. Laboratory testbeds and non-market valuation: The case of bidding behavior in a second-price auction with an outside option. Environmental and Resource Economics. 11 2004; 29(3):285–94. 10.1007/s10640-004-5264-z

[42] Buhrmester M, Kwang T, Gosling SD. Amazon's Mechanical Turk: A New Source of Inexpensive, Yet High-Quality, Data? Perspectives on Psychological Science. 03 2 2011; 6(1):3–5. 10.1177/1745691610393980 26162106

[43] Conard E. The Upside of Inequality: How Good Intentions Undermine the Middle Class: Portfolio; 2016.

[44] Broadberry S. Market services and the productivity race, 1850–2000: British performance in international perspective. Cambridge University Press; 2006.

[45] Seetharaman D. Facebook Posts Surge in Revenue as It Tackles User-Data Crisis. The Wall Street Journal, Dow Jones & Company 26 4 2018 Available from: https://www.wsj.com/articles/facebook-reports-higher-revenue-earnings-1524687694.

